# Type 1 Diabetes: A Chronic Anti-Self-Inflammatory Response

**DOI:** 10.3389/fimmu.2017.01898

**Published:** 2017-12-22

**Authors:** Matthew Clark, Charles J. Kroger, Roland M. Tisch

**Affiliations:** ^1^Department of Microbiology and Immunology, University of North Carolina at Chapel Hill, Chapel Hill, NC, United States; ^2^Lineberger Comprehensive Cancer Center, University of North Carolina at Chapel Hill, Chapel Hill, NC, United States

**Keywords:** autoimmunity, type 1 diabetes, immunoregulation, inflammation, T cells

## Abstract

Inflammation is typically induced in response to a microbial infection. The release of proinflammatory cytokines enhances the stimulatory capacity of antigen-presenting cells, as well as recruits adaptive and innate immune effectors to the site of infection. Once the microbe is cleared, inflammation is resolved by various mechanisms to avoid unnecessary tissue damage. Autoimmunity arises when aberrant immune responses target self-tissues causing inflammation. In type 1 diabetes (T1D), T cells attack the insulin producing β cells in the pancreatic islets. Genetic and environmental factors increase T1D risk by in part altering central and peripheral tolerance inducing events. This results in the development and expansion of β cell-specific effector T cells (Teff) which mediate islet inflammation. Unlike protective immunity where inflammation is terminated, autoimmunity is sustained by chronic inflammation. In this review, we will highlight the key events which initiate and sustain T cell-driven pancreatic islet inflammation in nonobese diabetic mice and in human T1D. Specifically, we will discuss: (i) dysregulation of thymic selection events, (ii) the role of intrinsic and extrinsic factors that enhance the expansion and pathogenicity of Teff, (iii) defects which impair homeostasis and suppressor activity of FoxP3-expressing regulatory T cells, and (iv) properties of β cells which contribute to islet inflammation.

## Introduction

Type 1 diabetes (T1D) is an autoimmune disease characterized by the chronic inflammation of the pancreatic islets of Langerhans (1–4). Islet inflammation is typically marked by infiltrating adaptive and innate immune effectors. Insulitis progresses over time and when a sufficient amount of β cell mass has been rendered nonfunctional and/or destroyed, hyperglycemic blood levels are achieved, and clinical diabetes established. The immune mechanisms mediating β cell autoimmunity are heterogeneous, as reflected by the nature of the islet infiltrate and the age of clinical onset. Nevertheless, T1D is generally viewed as a T cell-driven autoimmune disease, particularly for the more prevalent and aggressive type of T1D that develops in children and adolescents versus adults (5–17). A T cell-independent subtype of T1D, however, may also exist that is thought to be largely mediated by innate immune effectors (18, 19). The events leading to the loss of β cell-specific tolerance and chronic islet inflammation are complex, and influenced by both genetic and environmental factors (20–22).

Type 1 diabetes is polygenic with more than 20 insulin-dependent diabetes mellitus (*IDDM*) genetic loci identified that are associated with increased or decreased risk for T1D (23–28). The strongest genetic association is with the human leukocyte antigen locus (*IDDM1*), and particular class I and II haplotypes, consistent with a key role for T cells in T1D (29, 30). A number of genes regulating T, B, and innate cell immunobiology are also linked with T1D, as are genetic variants intrinsic to β cells, which deleteriously affect β cell function and/or responses to inflammation (31–37).

The identity and role of environmental factors in T1D are poorly understood. The most common hypothesis is that microbial infections initiate and/or exacerbate islet inflammation in genetically susceptible individuals (38, 39). For instance, T1D is associated with enteroviruses such as coxsackievirus B1 (40–44). Viral infection of β cells may result in direct cytolysis and/or elicit local inflammation that initiates and/or drives autoimmunity (45–47). The gut microbiota also has a profound regulatory effect on β cell autoimmunity (48, 49). In the nonobese diabetic (NOD) mouse, a spontaneous model of T1D, β cell destruction can be either promoted or prevented by changes in the composition of the gut microbiota (50, 51). Here bacterial components and metabolites are thought to impact the activation and/or differentiation status of innate and adaptive immune effectors. Longitudinal studies of at risk subjects also indicate a role for gut microbiota in human T1D (52–54).

**Figure 1:**
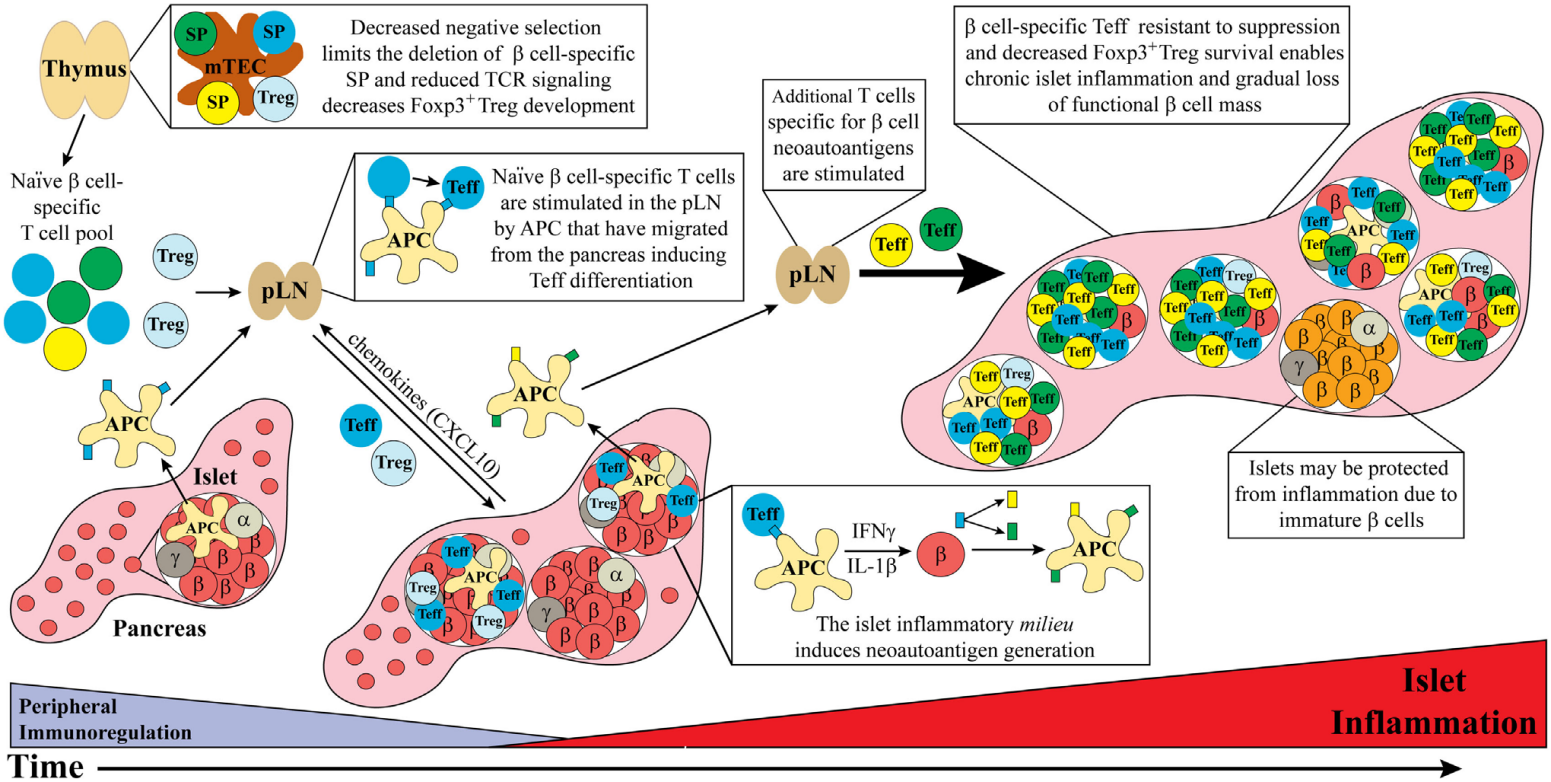
Dysregulated thymic and peripheral events culminate in chronic islet inflammation. In general, overt diabetes results from the gradual loss of functional insulin producing β cells due to the inflammatory environment driven by infiltrating self-reactive T cells and antigen-presenting cell (APC). Although β cell-specific T cell clones are detected in both healthy and type 1 diabetes (T1D) susceptible individuals, a number of factors promote T1D development in the latter population. Decreased efficiency of negative selection in the thymus, either due to altered tissue-specific antigen expression or due to T cell receptor (TCR) signaling, allows for the increased escape of β cell-specific T cell clones into the periphery. In addition, β cell-specific Foxp3^+^Treg development may also be suboptimal due to dysregulation of TCR signaling. In the periphery, β cell-specific T cells are stimulated in the pancreatic lymph nodes (pLN) by APC derived from the islets, leading to effector T cell (Teff) differentiation. These pathogenic Teff then infiltrate the islets and drive inflammation leading to reduced β cell function and/or survival. Not all islets are infiltrated potentially due to an immature phenotype and reduced autoantigen expression by β cells. Ongoing islet inflammation also leads to the generation of neoautoantigens either directly in β cells or during antigen processing by APC. The presentation of neoautoantigens within the pLN promotes the activation and expansion of additional Teff pools. These events amplify and drive a chronic state of islet inflammation leading to impaired functional β cell mass and clinical onset of T1D.

The T cell-related events that drive chronic islet inflammation in T1D stem from dysregulation of central and peripheral tolerance, alterations in self-antigen processing, and modified β cell responses (Figure 1). Here, we discuss how events critical for initiating and amplifying the development of T cell-mediated islet inflammation are regulated in NOD mice and human T1D.

## Thymic Origins of Diabetogenic T Cells: Setting the Stage for Islet Inflammation

The generation of an autoreactive T cell receptor (TCR) repertoire in the periphery is established in part by inefficient negative selection of anti-self-single positive thymocytes (SP) in the thymus (55, 56). Early in ontogeny negative selection is lax, resulting in increased escape of anti-self-SP (57–59). This temporal decrease in negative selection and elevated survival of β cell-specific clonotypes may help explain the predominance of T1D onset in childhood. With time, changes in thymic structural organization and maturation of thymic antigen-presenting cells (APC) leads to more efficient negative selection and increased death of autoreactive SP (57).

Key mediators of negative selection are medullary thymic epithelial cells (mTEC) and dendritic cells (DC). Notably, mTEC express and present several tissue-specific antigens (TSA) (60–63). Recognition of MHC-self-peptide complexes with increasing avidity/affinity results in elevated TCR signaling and SP apoptosis. Dysregulation of negative selection generates a peripheral pool of anti-self-T cells displaying increased avidity/affinity, and likely an enhanced “pathogenic potential.”

Parameters influencing the efficiency of negative selection both intrinsic and extrinsic to thymocytes have been linked to the development of β cell-specific T cells and T1D. Thymocyte intrinsic properties reported in NOD mice have included reduced SP sensitivity to apoptosis and altered double positive thymocyte differentiation to SP (64–67). In humans, TCR signaling needed to drive apoptosis of β cell-specific SP may be limited by a T1D-associated variant of the protein tyrosine phosphatase non-receptor 22 (*PTPN22*) gene (31, 32). PTPN22 is a negative regulator of TCR signaling, and elevated phosphatase activity by PTPN22 is predicted to reduce TCR signaling strength and diminish apoptosis induction in SP (68). An increase in PTPN22 activity may also limit thymic development of β cell-specific FoxP3-expressing regulatory CD4^+^ T cells (FOXP3^+^Treg), which is dependent on high(er) avidity/affinity recognition of self-peptide.

Thymocyte extrinsic factors that impact negative selection include aberrant expression of TSA in the medulla. The importance of thymic expression and presentation of TSA is readily evident in mice and humans deficient of the transcription factor autoimmune regulator (AIRE) (60, 69). Lack of AIRE, which drives expression of select TSA by mTEC, results in inefficient thymic negative selection and reduced development of tissue-specific Foxp3^+^Treg, leading to multiorgan autoimmunity in mice (70–74). Similarly, aberrant AIRE expression and function in humans results in the development of autoimmune polyendorinology candidiasis and ectodermal dysplasia (APECD) in which a variety of organs are targeted by T cells; notably a subset of APCED patients develop T1D (75, 76). Reduced AIRE expression has been reported in NOD mice, which reflects not only T1D development but also T cell-mediated inflammation of other tissues such as the thyroid, salivary, and lacrimal glands (77).

In human T1D, a strong genetic association is linked to the insulin encoding gene *INS2* found in *IDDM2* (78). Insulin is believed to be a key autoantigen driving human T1D, which is supported by studies in NOD mice (79–81). *INS2* is preceded by a variable number of tandem repeats (VNTRs). Individuals that have 26–63 VNTRs, associated with decreased thymic *INS2* expression, have an increased risk of developing T1D. In contrast, *INS2* expression is increased with VNTRs ranging between 140 and 210, which in turn is associated with a protective phenotype (82, 83). Reduced thymic insulin expression is expected to both limit negative selection and development of insulin-specific SP and FOXP3^+^Treg, respectively. Future studies are needed to directly demonstrate that thymic selection is dysregulated, and contributes to an expanded β cell-specific peripheral T cell pool in human T1D. Whether defects in thymic selection and development of β cell-specific T cells are necessary only early on or required throughout the disease process is another issue that needs to be tackled.

It is noteworthy that β cell-specific T cells are detected in the blood of healthy individuals, likely reflecting in part the reduced efficiency of thymic negative selection early in ontogeny. However, the phenotype of circulating β cell-specific T cells is distinct in T1D patients versus healthy subjects (84–89). The former exhibit mostly an effector/memory phenotype and expression of proinflammatory cytokines consistent with ongoing β cell autoimmunity (84–88). These findings indicate that in addition to the TCR repertoire, other factors contribute to the differentiation and expansion of diabetogenic effector T cells (Teff). For instance, the extent of tissue destruction and lethality of AIRE deficiency in mice is influenced by genotype with AIRE-deficient NOD versus C57BL/6 mice exhibiting more severe systemic autoimmunity (90, 91). Additionally, distinct TCR repertoires have been found in NOD mice in contrast to MHC matched C57BL/6 mice (92). Overall, dysregulation of thymic selection events in NOD mice acts as a precursor for islet inflammation.

## Extrinsic and Intrinsic Factors Promote Pathogenic Effector T Cells in T1D

The initiation of islet inflammation in NOD mice and humans is ill-defined. In NOD mice pancreatic remodeling shortly after birth is thought to play a key role starting the diabetogenic response (93, 94). Remodeling of the pancreas results in a wave of β cell apoptosis and release of antigens which are endocytosed by resident macrophages and DC (95). These APC then traffick to the draining pancreatic lymph nodes (pLN) to prime β cell-specific T cells and promote Teff differentiation (96, 97). Once established Teff migrate into the islets and mediate inflammation (97–99).

As alluded to above, shifts in the composition of the gut microbiota early in ontogeny are also believed to play a key role in regulating Teff differentiation in both mice and humans. Systemic release of microbiota-derived products can activate APC that in turn prime β cell-specific T cells providing an “environmental trigger” to incite T1D development (48). NOD mice in which the response to the microbiome is limited due to a deficiency in the Toll-like receptor adaptor protein MyD88, exhibit reduced β cell-specific Teff reactivity and diabetes incidence (50, 100). Strikingly, diabetes is prevented in NOD mice housed under germ-free conditions and inoculated with microbiota derived from MyD88-deficient animals (50), demonstrating that the microbiota also has a protective role in T1D. A less diverse gut microbiota in young individuals at risk for T1D is associated with progression to clinical diabetes (54). Changes in the gut microbiome have also been linked to the female bias of T1D in NOD mice (100). Interestingly, studies show that the lymphopenic environment in neonatal mice induces naïve T cells to rapidly expand and transition into a memory-like phenotype, that in turn is influenced by gut microbiota (101–104). Expansion of memory-like T cells, also seen in newborn humans, may enhance the pathogenic potential of the peripheral T cell pool and favor the development of autoimmunity in susceptible individuals.

Both CD4^+^ and CD8^+^ T cells are required for efficient β cell destruction in NOD mice (105). Islet CD8^+^ T cells primarily mediate β cell destruction by a cognate interaction involving perforin and granzyme B-, and Fas-Fas ligand-mediated killing (106, 107). On the other hand, islet CD4^+^ T cells drive β cell destruction in a bystander manner *via* secretion of proinflammatory cytokines. CD4^+^ and CD8^+^ T cells are also detected in the islets of diabetic subjects, with CD8^+^ T cells often predominating (6, 106). Several β cell autoantigens are recognized by the islet infiltrating T cells, and a number of these are similarly targeted in both the NOD and human diabetogenic responses including glutamic acid decarboxylase 65, proinsulin, insulin B chain, islet antigen-2, and islet-specific glucose-6-phosphatase catalytic subunit-related protein (108).

The majority of CD4^+^ and CD8^+^ T cells infiltrating the islets of NOD mice and T1D subjects exhibit a T helper 1 (Th1) effector phenotype, marked by IFNγ secretion (109). Increased Th17 cells are seen in the islets of NOD mice and the pLN of T1D subjects (109–111). The role of Th17 cells in mediating islet inflammation, however, is ill-defined. Elevated local levels of IFNγ are believed to establish a feed-forward loop that drives islet pathology. Based on NOD mouse studies, IFNγ secreted by islet CD4^+^ (and CD8^+^) Teff results in local upregulation of chemotactic cues that induce additional T, B, and innate cells to migrate into the islets, as well as promote islet retention of these effectors (109, 112). IFNγ also activates islet resident APC and stromal cells to elevate production of additional inflammatory mediators, such reactive oxygen species, which impair function and mediate β cell necrosis (107, 113, 114). Furthermore, IFNγ in the context of IL-1β and TNFα induces β cell apoptosis (113, 114).

Another proinflammatory cytokine thought to contribute to islet inflammation is IL-21 which is elevated in T1D patient serum (115). Notably, the murine IL-21 gene is located in the *Idd3* locus and IL-21 receptor (R) deficiency prevents T1D in NOD mice (116). CD4^+^ T follicular helper cells, which are increased in the pLN of NOD mice, are the primary source of IL-21 (112, 117–119). IL-21 has a critical role in supporting B cell development and antibody production. B cells, serving as APC, are required for efficient β cell destruction in NOD mice and likely in human T1D (117, 118, 120, 121). IL-21 also enhances maintenance of CD8^+^ Teff by preventing exhaustion during chronic inflammation (122, 123). Interestingly, the pathogenicity of β cell-specific CD8^+^ T cells is dependent on IL-21R expression (124, 125).

Defects intrinsic to Teff are also thought to facilitate chronic islet inflammation. Variants of the *CTLA4* gene are linked to T1D susceptibility in both NOD mice (*Idd5.1*) and human T1D (*IDDM12*) (33, 126). CTLA-4 which binds to the costimulatory molecules CD80 and CD86 expressed on APC, is a negative regulator of T cell activation and proliferation (34). Polymorphisms in the human *CTLA4* gene region are associated with reduced mRNA levels and a decrease in expression of the soluble (s) CTLA-4 isoform (33, 34, 126). sCTLA-4 also negatively regulates TCR signaling (33, 34, 126). Reduced expression of CTLA-4 and sCTLA-4 is expected to facilitate expansion of β cell-specific T cells. This scenario is consistent with the exacerbated β cell autoimmunity seen in NOD mice expressing a diabetogenic TCR transgene and lacking CTLA-4 expression (127). Noteworthy is that both NOD-derived and human T1D Teff also exhibit reduced sensitivity to Foxp3^+^Treg-mediated suppression (128, 129). In sum, the culmination of a variety of extrinsic and intrinsic factors enables Teff to expand, persist, and in turn amplify islet inflammation.

## Defects in the Foxp3^+^Treg Pool Contribute to T1D

In addition to Teff that are resistant to regulatory mechanisms that limit expansion and function, evidence indicates that the Foxp3^+^Treg pool is compromised in T1D (130, 131). Here, dysregulation of Foxp3^+^Treg homeostasis is thought to permit preferential differentiation and expansion of pathogenic β cell-specific Teff. Foxp3^+^Treg have an essential role in regulating immune homeostasis and reactivity to self (132–135). The lack of thymic development of Foxp3^+^Treg due to deficient expression or function of the FoxP3 transcription factor, results in systemic autoimmunity in both mice and humans. Foxp3^+^Treg mediate suppression of T cells and other immune effectors *via* multiple mechanisms including cell-contact dependent suppression, and secretion of anti-inflammatory cytokines and mediators such as IL-10, TGFβ1, and IL-35, and adenosine, respectively (136). Foxp3^+^Treg also function as an “IL-2 depot” to deprive Teff of IL-2 needed for expansion (136). The latter is mediated by constitutive expression of CD25, the α subunit of the IL-2R (136). Therefore, Foxp3^+^Treg, expressing the high affinity IL-2R, are able to out compete Teff for IL-2, which transiently express high affinity IL-2R.

IL-2 is essential for Foxp3^+^Treg homeostasis, expansion, and function (136). Unlike conventional T cells, Foxp3^+^Treg do not produce IL-2 due to FoxP3-mediated negative regulation of *Il2* transcription. Therefore, Foxp3^+^Treg are dependent on T cells and DC as IL-2 sources (136). This dependency is thought to enable Foxp3^+^Treg to more readily sense and respond to ongoing inflammation. Accordingly, defects in the IL-2/IL-2R axis have been described in the NOD model and human T1D (137–142). In NOD mice, an *Il2* variant located in *Idd3* results in reduced levels of IL-2 expression by Teff, and impaired survival and function of islet resident Foxp3^+^Treg (130, 143). Increased levels of proinflammatory cytokines, such as IFNγ and IL-6 that downregulate FoxP3 expression may also promote dedifferentiation of islet Foxp3^+^Treg into a Teff-like subsets (144, 145). These events lead to a progressive loss of islet Foxp3^+^Treg suppression, thereby “releasing the brakes” and favoring pathogenic Teff expansion.

The frequency of FOXP3^+^Treg found in blood is largely unaffected in T1D subjects (140, 141, 146–148). However, FOXP3^+^Treg from T1D subjects exhibit reduced suppressor function measured *in vitro* (128, 129). This aberrant activity is linked to T1D risk variants of *IL2RA* (CD25) and *PTPN2*, a phosphatase involved in IL-2R signaling (149). Notably, FOXP3^+^Treg expressing these variants display reduced IL-2R signaling that in turn correlates with limited suppressor activity (149, 150). Defects in IL-2R signaling have led to clinical studies testing whether low-dose IL-2 therapy enhances the FOXP3^+^Treg pool in T1D subjects (149, 151). This approach has been effective in preventing and/or reversing diabetes in NOD mice by increasing the number and function of islet Foxp3^+^Treg (149, 152). One key question not addressed is the specificity of FOXP3^+^Treg in T1D subjects. Reduced thymic development of β cell-specific FOXP3^+^Treg, as discussed above, would be expected to limit the “anti-diabetogenic” effects of the peripheral FOXP3^+^Treg pool.

## Amplifying the Pathogenic Effector T Cell Response *via* Neoautoantigens

Recent findings have demonstrated that the proinflammatory *milieu* of the islets promotes processing of “neoautoantigens” (153). Importantly, these neoautoantigens are only found in the periphery so that corresponding T cell clonotypes, not deleted in the thymus and possibly expressing high affinity TCR, can be recruited into the inflammatory response. Neoautoantigens are generated *via* posttranslational modifications (PTM), such as deamidation by tissue transglutaminase (tTG) (153). PTM can occur during APC antigen processing or directly in β cells (154). tTG-dependent deamidation of a proinsulin C-peptide for instance is detected in both human DC and islets under inflammatory conditions (154). Notably the resulting modified peptide is recognized by CD4^+^ T cells derived from T1D subjects. The MHC binding and in turn T cell stimulatory properties of peptides can also be enhanced by deamidation (155).

Neoautoantigens consisting of hybrid peptides have recently been identified. In NOD mice hybrid insulin peptides are generated *via* covalent crosslinking of a proinsulin C peptide with peptides derived from naturally occurring cleavage products produced in the β cell secretory granules (156). In addition to ongoing inflammation, PTM occurs *via* endoplasmic reticulum stress, which can be induced in β cells by the normal physiological demands associated with high levels of insulin secretion (157). Therefore, it is possible that β cell neoautoantigens in addition to amplifying inflammation, play a role in initiating the diabetogenic response. Neoautoantigens are also generated at a transcriptional level. A mutation in the open reading frame of insulin mRNA generates a neoautoantigen that stimulates CD8^+^ T cells from T1D subjects causing β cells lysis *in vitro* (158). Alternative RNA splicing may be another mechanism leading to neoautoantigen expression, particularly since ~30% of genes in inflamed β cells undergo aberrant alternative splicing (159). In sum, β cell neoautoantigens serve as *bona fide* targets of pathogenic CD4^+^ and CD8^+^ T cells. The breadth of the peptidome of neoautoantigens produced and presented, and the properties of neoautoantigen-specific T cells, in terms of frequency, avidity/affinity, subset phenotype (e.g., pathogenic versus regulatory), and overall contribution to islet inflammation require further investigation.

## β Cell-Intrinsic Properties That Regulate Islet Inflammation

Studies have demonstrated that intrinsic properties of β cells also influence islet inflammation. For instance, CXCL10 is produced by β cells although the role of this chemokine in disease is controversial (160). CXCL10 regulates migration of CXCR3 expressing Teff and Foxp3^+^Treg into the islets (97–99, 161). Overexpression of CXCL10 in β cells accelerates T1D progression, and antibody blockade of CXCL10 prevents Teff migration into the islets of NOD mice (97–99, 161). On the other hand, *Cxcr3* deficiency accelerates T1D by reducing islet resident Foxp3^+^Treg (162, 163). Therefore, depending on the context, β cells may affect inflammatory and immunoregulatory events *via* CXCL10 production. The chemokine CCL2 is also secreted by β cells, and over-expression of ectopic CCL2 recruits tolerogenic CCR2-expressing DC and blocks T1D progression in NOD mice (164). Interestingly, NOD APC shows defective migration in response to CCL2, and human T1D patients have reduced serum levels of CCL2 (164–166). Additionally, β cells produce CXCL1 and CXCL2 that recruit CXCR2-expressing neutrophils to the islets, which contribute to stimulating β cell-specific T cell reactivity (167, 168). Overall, β cell produced chemotactic cues regulate the progression of the diabetogenic response.

Along with chemokines, β cells secrete the cytokine IL-1β, which at low levels promotes β cell proliferation, and enhances production of CCL2, CXCL1, CXCL2, and insulin (169, 170). However, IL-1β also primes leukocyte effector-mediated inflammation, and as noted above, IL-1β in the context of TNFα, and/or IFN-γ induces β cell apoptosis *in vitro* (113, 114). Notably, glucagon-secreting α cells also produce IL-1β, indicating that other islet resident endocrine cells may also contribute to local inflammation (171, 172). Islet inflammation also induces upregulation of MHC class I and II on β cells to further increase β cell immunogenicity (173). Interestingly, a subpopulation of β cells have been identified which under inflammatory conditions acquires resistance to immune-mediated destruction in NOD mice (174). The latter is associated with a more immature β cell phenotype coupled with reduced expression of autoantigens and upregulation of immunomodulatory molecules such as PD-L1, an inducer of T cell exhaustion. A similar phenotype is seen for human β cells (174). Therefore, β cells not only contribute to islet inflammation but also adapt under the inflammatory conditions in order to persist. A better understanding of the events regulating this dichotomy has important implications for the treatment of T1D patients *via* β cell replacement strategies for instance.

## Summary

Type 1 diabetes is complex involving genetic, epigenetic and environmental factors that influence adaptive and innate effector cell populations, which ultimately culminate in pathological, chronic islet inflammation (Figure 1). The heterogeneity associated with human T1D and in turn the nature of islet inflammation is expected to reflect the genotype of the individual, and type of environmental insult(s) encountered (20–22). These factors dictate which immune effectors are the key drivers of pathology, the pace of disease progression, and the degree of β cell dysregulation and/or death. We propose that the rapid and aggressive T1D seen early in life is marked by a broad β cell-specific TCR repertoire with increased avidity/affinity due to insufficient negative selection (57–59). This is coupled with β cell-specific Teff that are insensitive to peripheral tolerance inducing events, β cell-specific FOXP3^+^Treg with impaired suppressor activity, and β cells which readily promote islet inflammation (128, 129, 147, 169, 170). Robust inflammation leads to increased β cell neoautoantigen production further amplifying the kinetics and overall inflammatory response (153, 154, 156, 158). Under these “ideal” conditions, early onset T1D develops. On the other hand, in individuals with only a partial complement of these key “disease components,” islet inflammation is less robust and the kinetics of T1D onset proportionately delayed. Defining the events driving early versus late(r) T1D onset is critical for a better understanding of how islet inflammation is regulated in humans. The latter is also important for developing rational and effective immunotherapies to prevent and/or treat T1D. Devising strategies to enhance thymic negative selection early in ontogeny for instance, would be expected to purge the diabetogenic TCR repertoire to prevent T1D. Indeed, approaches are currently being studied to manipulate thymic negative selection in the context of cancer treatment by expanding the T cell repertoire specific for self-tumor antigens (175–177). Altering the gut microbiome early in life may also prove to be an effective strategy to limit expansion of the anti-self-T cell repertoire and establish robust immunoregulation in the periphery. Administration of β cell neoautoantigens may augment the efficacy of antigen-based immunotherapies to block disease progression at later stages of T1D (157). Depending on the mode of administration, β cell neoautoantigens can be used to target the corresponding clonotypes by tolerizing pathogenic Teff and/or inducing/expanding FOXP3^+^Treg. In view of the heterogeneity in the immunopathology of T1D, it is very likely these approaches and others currently being studied will need to be combined to effectively suppress the chronic islet inflammation and β cell autoimmunity long term.

## Author Contributions

MC, CJK, and RMT contributed to the preparation of the review article.

## Conflict of Interest Statement

The authors have no personal, professional, or financial relationships that are considered to be a conflict of interest.
